# Long-Term Gene Therapy Causes Transgene-Specific Changes in the Morphology of Regenerating Retinal Ganglion Cells

**DOI:** 10.1371/journal.pone.0031061

**Published:** 2012-02-08

**Authors:** Jennifer Rodger, Eleanor S. Drummond, Mats Hellström, Donald Robertson, Alan R. Harvey

**Affiliations:** 1 Experimental and Regenerative Neuroscience, School of Animal Biology, The University of Western Australia, Perth, Australia; 2 School of Anatomy and Human Biology, The University of Western Australia, Perth, Australia; 3 Discipline of Physiology, School of Biomedical and Biomolecular Sciences, The University of Western Australia, Perth, Australia; Universidade Federal do Rio de Janeiro, Brazil

## Abstract

Recombinant adeno-associated viral (rAAV) vectors can be used to introduce neurotrophic genes into injured CNS neurons, promoting survival and axonal regeneration. Gene therapy holds much promise for the treatment of neurotrauma and neurodegenerative diseases; however, neurotrophic factors are known to alter dendritic architecture, and thus we set out to determine whether such transgenes also change the morphology of transduced neurons. We compared changes in dendritic morphology of regenerating adult rat retinal ganglion cells (RGCs) after long-term transduction with rAAV2 encoding: (i) green fluorescent protein (GFP), or (ii) bi-*cis*tronic vectors encoding GFP and ciliary neurotrophic factor (CNTF), brain-derived neurotrophic factor (BDNF) or growth-associated protein-43 (GAP43). To enhance regeneration, rats received an autologous peripheral nerve graft onto the cut optic nerve of each rAAV2 injected eye. After 5–8 months, RGCs with regenerated axons were retrogradely labeled with fluorogold (FG). Live retinal wholemounts were prepared and GFP positive (transduced) or GFP negative (non-transduced) RGCs injected iontophoretically with 2% lucifer yellow. Dendritic morphology was analyzed using Neurolucida software. Significant changes in dendritic architecture were found, in both transduced and non-transduced populations. Multivariate analysis revealed that transgenic BDNF increased dendritic field area whereas GAP43 increased dendritic complexity. CNTF decreased complexity but only in a subset of RGCs. Sholl analysis showed changes in dendritic branching in rAAV2-BDNF-GFP and rAAV2-CNTF-GFP groups and the proportion of FG positive RGCs with aberrant morphology tripled in these groups compared to controls. RGCs in all transgene groups displayed abnormal stratification. Thus in addition to promoting cell survival and axonal regeneration, vector-mediated expression of neurotrophic factors has measurable, gene-specific effects on the morphology of injured adult neurons. Such changes will likely alter the functional properties of neurons and may need to be considered when designing vector-based protocols for the treatment of neurotrauma and neurodegeneration.

## Introduction

Replication-deficient viral vectors such as recombinant adeno-associated virus (rAAV) are increasingly being used to introduce ‘therapeutic’ genes into neural cells, a method that allows targeted supply of neuroprotective and/or growth-promoting molecules to the injured or degenerating CNS [1]–[5]. In the eye, vitreal injection of rAAV serotype 2 (rAAV2) or other viral vectors encoding growth factors increases retinal ganglion cell (RGC) survival and axonal regeneration after optic nerve (ON) injury [6]–[9].

The gene therapy approach holds much promise for the treatment of neurodegenerative diseases and retinal dystrophies [10]–[13], as well as potentially enhancing repair after neurotrauma [14]. However, what is not yet clear is the extent to which long-term constitutive expression of transgenes changes the structure and function of transduced neurons. This is especially relevant when using genes that encode, for example, secretable neurotrophic factors because these peptides are known to alter dendritic architecture, synaptic density and plasticity, cause down-regulation of cognate receptors and modulate activity of signaling molecules [15]–[19]. Thus persistent over-expression of some transgenes may alter local circuitry and neuronal responsiveness to endogenous neuroactive factors [1], [20], [21].

We therefore set out to determine whether soma size and dendritic architecture is altered after prolonged rAAV2 vector transduction, and whether any such changes depend on the type of gene that is introduced into CNS neurons. Because rAAV based gene therapy will potentially be used in post-injury as well as neurodegenerative conditions, we chose to use our established visual system regeneration model to quantitatively analyze changes in adult rat RGCs that had been axotomized and then induced to regenerate. RGC viability and long-distance axonal regeneration was promoted by grafting an autologous peripheral nerve (PN) segment onto the cut ON [22], [23]. Four vectors were tested: rAAV2-GFP alone, and bi-*cis*tronic rAAV2 vectors encoding either brain-derived neurotrophic factor (rAAV2-BDNF-GFP), a secretable form of ciliary neurotrophic factor (rAAV2-CNTF-GFP), or a non-secreted protein growth-associated protein 43 (rAAV2-GAP43-GFP). A saline-injected control group was also included. BDNF, CNTF, and GAP-43 have all been shown to influence adult RGC viability and axonal regeneration [23], and the impact of each of these genes when encoded in rAAV vectors has been documented previously in rat PN-ON graft studies [8], [9].

Five to eight months after the initial surgery, regenerated RGCs were identified by retrogradely labeling them with fluorogold (FG) injected into the distal end of each PN graft. Living retinas were subsequently removed, wholemounted, and regenerated FG positive (^+^) RGCs were intracellularly injected with Lucifer Yellow (LY). Expression of GFP in all rAAV vectors permitted identification of transduced, regenerated RGCs. GFP^+^ and non-transduced GFP negative (^−^) RGCs that had regrown an axon into the PN graft were filled. After immunoprocessing for LY, soma size and dendritic morphology were analyzed and quantified using Neurolucida software. We observed gene-specific changes in the morphology of identified, regenerating adult RGCs after long-term rAAV2 therapy, not only in transduced RGCs but also in non-transduced RGC populations. Furthermore, some changes appeared to be subtype specific, seen only in large, type RI-like RGCs.

## Results

### Impact of transgenes on RGC morphology

**Figure 1:**
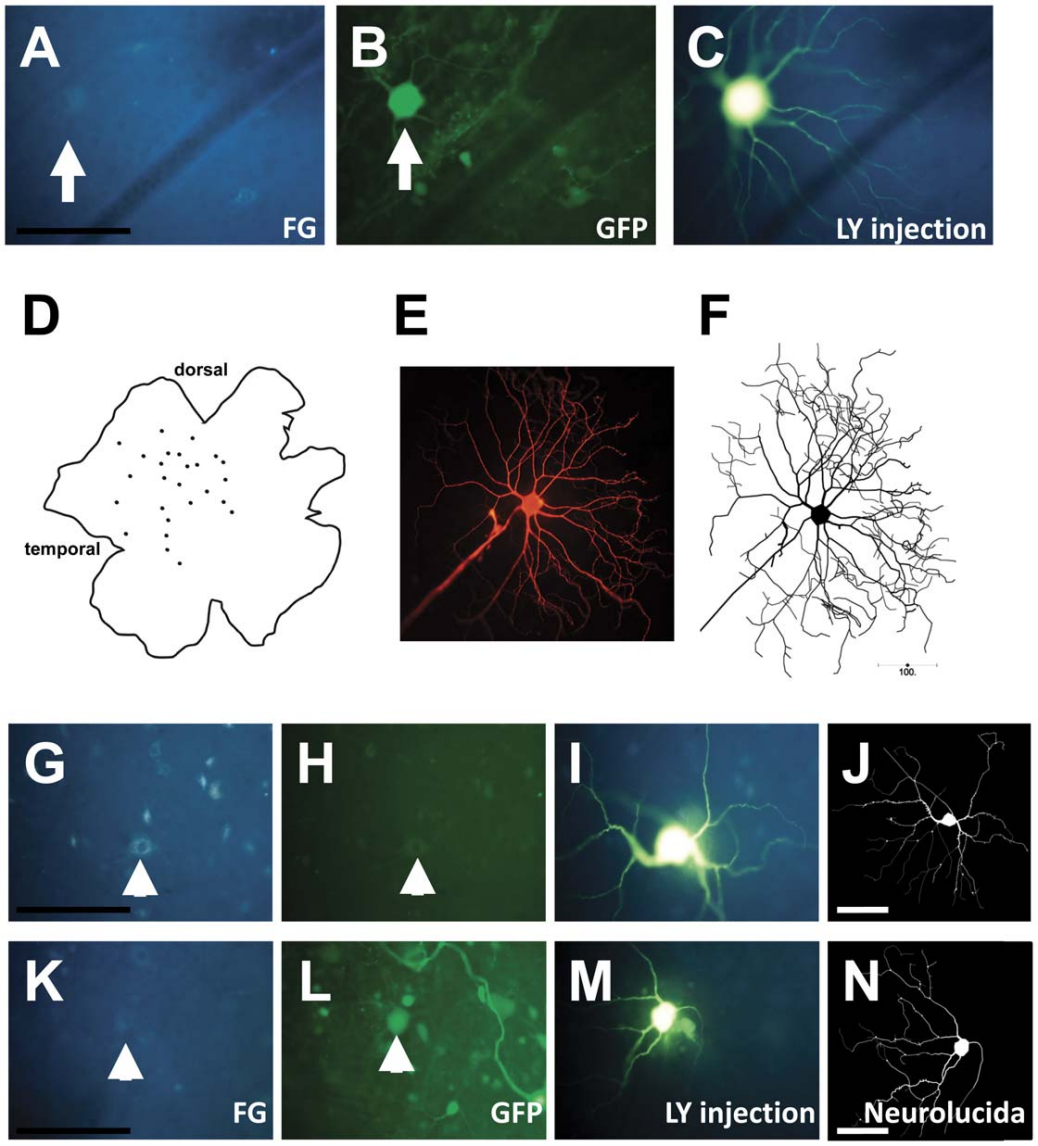
Injection, tracing and identification of individual RGCs from rAAV2-injected retinae. Photomicrographs and Neurolucida-traced images showing the procedure for labeling and identifying regenerating retinal ganglion cells (RGCs). Regenerating RGCs were first identified based on retrograde labeling (Fluorogold; A), and transduced cells were based on GFP expression (GFP^+^ RGC is shown in B). After filling the dendrites with Lucifer yellow (C), the RGC was again photographed. This procedure was repeated on 20–50 RGCs per retina (D). The visualization of dendritic architecture was further enhanced with Lucifer yellow immunohistochemistry, and individual cells with complete fills (E) were traced using Neurolucida software. The Neurolucida trace (F) was compared to images of the cells taken immediately after the Lucifer yellow injection (C) to allow each cell to be classified as transduced (GFP^+^) or as a non-transduced “bystander” neuron (GFP^−^). G–N: Representative images of RGCs that were retrogradely labeled with Fluorogold (G, K), identified as GFP^−^ (H) or GFP^+^ (L), injected with Lucifer yellow (I,M) and traced using Neurolucida software (J,N). Scale bars: 100 µm.

FG^+^ RGCs that had regenerated an axon to the distal end of PN grafts were identified and photographed under UV light (Fig. 1A, G) and also at 488 nm to determine whether the RGCs were transduced (GFP^+^) or non-transduced (GFP^−^; Fig. 1B, K, L). In all groups, transduced RGCs were most frequently seen in temporal retina, in the vicinity of the initial rAAV2 vitreal injection (Fig. 1D) [14], [24]. RGCs were injected iontophoretically with LY and a photograph taken under 488 nm (Fig. 1C, I, M) for subsequent identification of individual RGCs from immunolabelled and Neurolucida traces (Fig. 1E, F, J, N). This procedure was repeated on 20–50 RGCs per retina (Fig. 1D). The number of regenerated FG^+^ RGCs that were analyzed in each control or vector group is shown in Table 1. For clarity, throughout the text for each of the 4 vectors used in this study we will denote transduced and non-transduced (nt) FG^+^ RGCs as GFP/ntGFP, BDNF/ntBDNF, CNTF/ntCNTF or GAP43/ntGAP43 respectively.

**Table 1:** Number of RGCs analyzed in each control and experimental group for all RGCs, and for those identified as Type RI cells.

		All cells	Type RI
**Saline**		68	17
**BDNF**		73	40
	**ntBDNF**	14	7
	**BDNF**	59	33
**CNTF**		117	42
	**ntCNTF**	62	22
	**CNTF**	55	20
**GAP43**		63	19
	**ntGAP43**	16	4
	**GAP43**	47	15
**GFP**		54	20
	**ntGFP**	14	5
	**GFP**	39	15
**Total analysed**		**375**	**138**
**No axon/FG-**		66	
**Incomplete fill**		59	
**Grand Total**		**500**	

**Figure 2:**
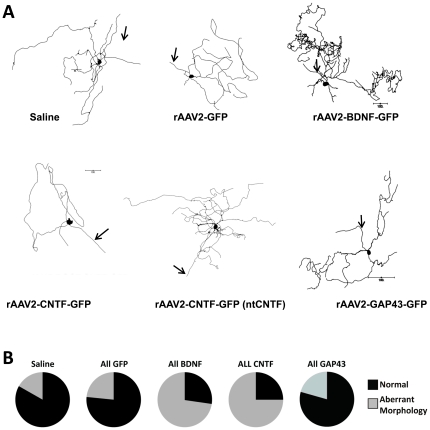
Increased prevalence of RGCs with abnormal morphology in retinae injected with rAAV2-BDNF-GFP and rAAV2-CNTF-GFP. A: Representative Neurolucida traces showing retinal ganglion cells (RGCs) with abnormal morphology. The term ‘aberrant morphology’ was used to describe RGCs with one extremely long dendrite that was exceptionally tortuous or abnormally sparse or asymmetric. Arrows indicate axons. The rAAV2 treatment group is indicated under each cell; nt = non-transduced RGC. B: Pie charts showing frequency distribution of RGCs with aberrant morphology in control and experimental groups.

In some vector groups, a significant proportion of the 375 fully analyzed RGCs possessed one or more highly abnormal dendritic morphologies, including either very sparse dendrites or unusually tangled processes (Fig. 2A). The proportion of RGCs with such abnormal dendritic morphologies was, compared to saline, not significantly different in rAAV2-GFP and rAAV-GAP43-GFP groups, but was significantly increased in the rAAV2-BDNF-GFP and rAAV2-CNTF-GFP injected groups (X_2_ = 130; p<0.0001; Fig. 2B).

### Discriminant analysis (summarized in Table 2)

**Table 2:** Summary of main dendritic morphological changes of long-term gene therapy affected retinal ganglion cells (RGCs) compared to appropriate GFP transduced or non-transduced control groups.

	ALL RGCS		ALL RGCS	TYPE I-LIKE RGCS
**BDNF**	Increased soma area (P<0.0001), longer dendrites (p = 0.02), larger dendritic field (p = 0.003), increased total and average nodal distance (p = 0.008; p = 0.03). Sholl analysis: denser dendrites. Deeper stratification (p = 0.004). Increased prevalence of aberrant morphology.	**BDNF transduced**	Increased soma area (p<0.0001), increased field size (p = 0.003), deeper stratification (p = 0.049).	Increased soma area (p = 0.04)
		**BDNF non-transduced**	No change	Increased soma area (p = 0.04), increased field area (trend: p = 0.063), deeper stratification (p = 0.01).
**CNTF**	Increased soma area (p<0.0001). Sholl analysis: denser dendrites. Deeper stratification (p = 0.003). Increased prevalence of aberrant morphology.	**CNTF transduced**	Increased soma area (p<0.0001), deeper stratification (p = 0.02).	Increased soma area (p = 0.0002), deeper stratification (p = 0.02).
		**CNTF non-transduced**	Increased soma area (p<0.0014), deeper stratification p = 0.02	Increased soma area (p = 0.001), reduced complexity (nodes (p = 0.006), branch order (p = 0.03), increased terminal/nodal distance (total and mean: p = 0.05), deeper stratification (p = 0.03).
**GAP43**	Increased complexity (fractal count: p = 0.02). Increased dendritic density (p = 0.02). Deeper stratification p = 0.01	**GAP43 transduced**	Increased complexity (fractal count: p = 0.04). Deeper stratification (p = 0.002).	Increased tortuosity (p = 0.01). Deeper stratification (p = 0.0009)
		**GAP43 non-transduced**	Increased dendritic density (p = 0.03)	No change

To quantify the morphological changes in regenerate RGCs induced by transgene expression, we applied multivariate statistical analysis to take into account all of the 15 parameters that were measured using Neurolucida (see Materials and Methods for further detail). We used discriminant analysis, a statistical technique used for differentiating groups using multiple quantitative variables [25]. The analysis extracts canonical scores, which represent a transformation of the original measurements into an expression of maximal differences between groups. Where significant differences were detected between the canonical scores of experimental and control groups, we then performed post-hoc analysis of the morphological measurements to determine the nature of these differences.

**Figure 3:**
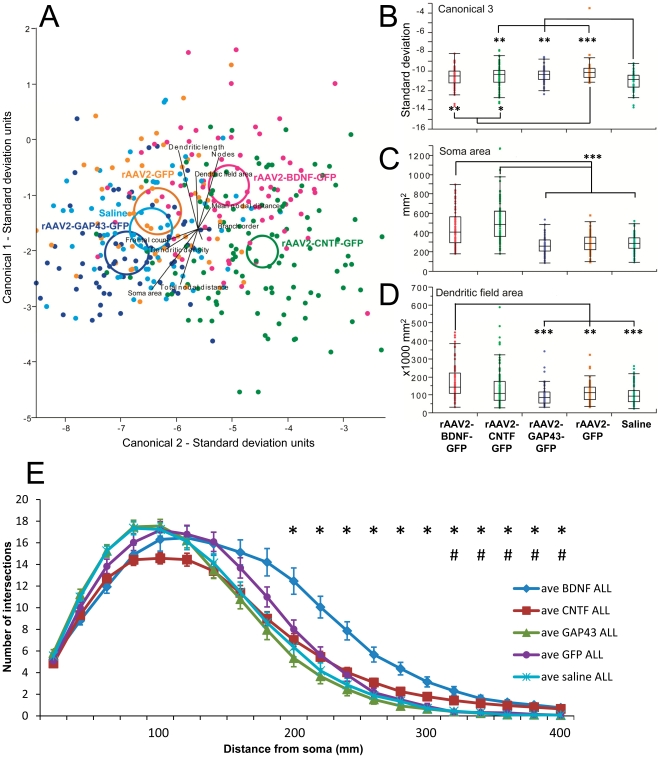
Evidence for morphological differences in RGCs from retinae injected with rAAV2 encoding different transgenes. A: Plot showing canonical scores 1 (Y axis) and 2 (X axis) from a multivariate discriminant analysis of dendritic morphology of all retinal ganglion cells (RGCs). Plots show the first two canonical scores that together represent more than 80% of the variance. Axes represent units of standard deviation. Circles represent the 95% confidence region to contain the true mean of the treatment groups. Black lines show the coordinate direction (i.e. morphological parameters measured in Neurolucida) in canonical space. Note that the length of the lines is not representative of effect size due to the multidimensional nature of the analysis. B–D: Box plots showing median and quartiles for selected morphological parameters that were significantly different between treatment groups. B: values for the third canonical score which accounts for the significant difference between Saline and rAAV2-GFP injected groups. Means for soma area (C) and dendritic field area (D) are shown for all treatment groups. * p<0.05; **p<0.001; *** P<0.0001. E: Sholl analysis of all RGCs by treatment group. Error bars = standard error of the mean. Asterisk (*) indicates significant (p<0.05) difference between rAAV2-BDNF-GFP and GFP; # indicates significant (p<0.05) difference between rAAV2-CNTF-GFP and GFP.

Discriminant analysis of the 5 treatment groups confirmed significant differences between all treatment groups. Most of the differences relative to control groups (saline and rAAV2-GFP injected retinae) were contained in canonical scores 1 and 2 (Fig. 3A). In addition, canonical score 3 demonstrated that RGCs from rAAV2-GFP transduced retinae differed from saline injected controls (p<0.0001; Fig. 3B). Because the canonical scores represent a transformation of the data from the morphological parameters they cannot be attributed to specific morphological differences. We therefore performed ANOVA and posthoc analysis on all of the measurements to identify how GFP expression affected morphology. However, no individual parameter was found to be responsible for this difference either in transduced or non-transduced cells, suggesting the effects of GFP on RGC morphology were relatively minor. Nonetheless in all of the following analyses we compared treatment groups to rAAV2-GFP controls and not to saline injected controls.

In rAAV2-BDNF-GFP injected retinae, the soma size of all LY-injected, regenerating RGCs was significantly increased compared to LY labeled, FG^+^ RGCs in rAAV2-GFP controls (P<0.0001; Fig. 3C). In addition, dendrites were longer (p = 0.02), field size was larger (p = 0.003; Fig. 3D), and total and mean nodal distances were longer (p = 0.008 and p = 0.003 respectively). When transduced and non-transduced RGCs were analyzed separately, most of the changes could be attributed to transduced BDNF neurons, which had larger somata (P<0.0001) and larger field size (p = 0.03) compared to their GFP transduced counterparts. No significant differences were observed in regenerate ntBDNF compared to ntGFP RGCs.

In rAAV2-CNTF-GFP injected retinae, the only difference compared to rAAV-GFP injected retinae was a significant increase in the soma size of RGCs (p<0.0001). The increase was observed in both CNTF and ntCNTF RGCs compared to GFP (p<0.0001) and ntGFP (p = 0.0014) RGCs respectively. In addition, CNTF RGCs had significantly larger soma size compared to ntCNTF RGCs (Table 2; p = 0.0085).

For RGCs in rAAV2-GAP43-GFP injected retinae, the fractal count [26] was significantly increased compared to rAAV2-GFP controls (p = 0.02), and dendrite density was also increased (p = 0.02). Analysis of transduced versus non-transduced neurons revealed that the difference in fractal count was found only in transduced GAP43 RGCs (p = 0.04), whereas an increase in dendritic density was found only in ntGAP43 RGCs (p = 0.03).

### Sholl analysis

To further characterize the morphological changes induced in RGCs by the different transgenes, we also performed a Sholl analysis, the most commonly used method to measure dendritic field density and structure [27]. The method assesses the distribution of dendrites as a function of eccentricity using the cell body as the centre. Analysis of the total cell population revealed that RGCs in rAAV2-BDNF-GFP and rAAV2-CNTF-GFP injected retinae had significantly denser dendrites from 200 µm to 400 µm (BDNF) and from 320 µm to 400 µm (CNTF) distal from the cell body compared to rAAV2-GFP controls (Fig. 3E). No significant differences were detected when transduced and non-transduced RGCs were analyzed separately.

### Assessment of morphological changes in one putative RGC subtype

The analysis above demonstrates that each transgene had a distinct and significant impact on dendritic morphology of RGCs. However, it is well established that there are multiple different subtypes of RGCs within the rat retina with characteristic morphologies [28], [29]. It is therefore desirable to analyze these subtypes separately to confirm that morphological differences between subtypes have not masked differences due to transgene expression. However, unlike in mouse, in which there is a correspondence of specific molecular markers with RGC cell subtype [30], [31], in rat there is no definitive method of classifying all RGC subtypes independent of morphology. The only phenotypic marker consistently used in rat RGCs is melanopsin, which is expressed in about 3% of RGCs. However, in the specific context of RGC axonal regeneration into a PN-ON graft, murine melanopsin expressing RGCs do not regenerate more frequently than other RGCs [32], thus in our rat study we would expect only about 10–15 regenerate RGCs to be melanopsin positive, clearly too few for any justifiable analysis.

Nonetheless, there is consensus across many studies that rat RGCs can be effectively classified into three or four main subtypes based on cell body size, number of dendrites, dendritic field size and stratification [29], [33]–[36]. Most importantly, this morphological classification has also been applied to RGCs in peripheral nerve grafted animals [37], [38], suggesting that regenerating RGCs maintain core structural features of their subtype despite injury-induced alterations in dendritic architecture [39]. Consistent with this, the outcome of our whole population analysis of the 375 LY-labeled RGCs did not reveal any overall change in the number of primary dendrites emanating from RGCs in any vector group. Using only morphological criteria, the best consensus across all studies is the identification of the RGC 1 subtype, known as RI in regenerating RGCs [37], [38]. These cells have a large soma, 4–6 primary dendrites and large dendritic field area with a very typical dendritic branching pattern [29], [36]. These same criteria identify type RI RGCs following a PN graft [37], [38]. Types 2 and 3 have smaller cell bodies than type 1 cells, fewer than 3 primary dendrites, and are differentiated primarily by the number of branch points but this measure becomes unreliable in regenerating RGCs in which branching density is greatly reduced [37].

**Figure 4:**
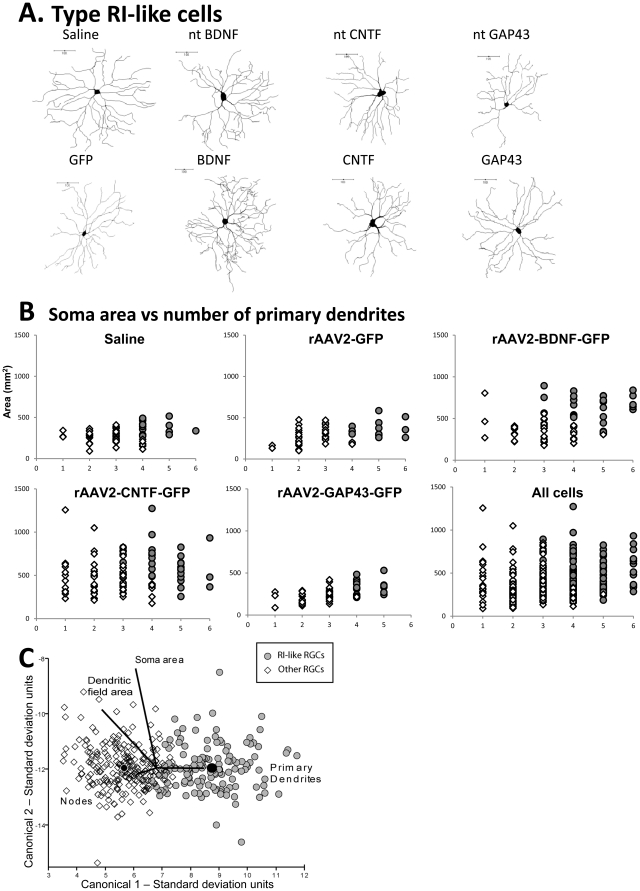
Classification of RI-like RGCs. A: Neurolucida traces of representative RI-like retinal ganglion cells (RGCs) from control and experimental rAAV2 groups. Transduced and non-transduced (nt) FG^+^ RGCs in the 4 rAAV2 groups are labeled as GFP/ntGFP, CNTF/ntCNTF, BDNF/ntBDNF or GAP43/ntGAP43 respectively. B: Clustering of transduced and ntRI-like RGCs based on the number of primary dendrites (X-axis) and soma area (Y axis) in control and experimental groups. RI-like RGCs are denoted by grey circles and remaining cells by white diamonds. C: Plot showing canonical scores 1 (X axis) and 2 (Y axis) from a multivariate discriminant analysis of dendritic morphology of RI-like RGCs and “other” RGCs. Plots show the first two canonical scores that together represent more than 80% of the variance. Axes represent units of standard deviation. Solid black circles represent the 95% confidence region to contain the true mean of the group. Black lines show the coordinate direction (i.e. morphological parameters measured in Neurolucida) in canonical space. Note that the length of the lines is not representative of effect size due to the multidimensional nature of the analysis.

Taking this previous literature into account, we conservatively assessed whether it was possible to identify RI-like RGCs within our general population using the number of primary dendrites, cell body size and typical dendritic branching pattern as the essential criteria. We identified all RGCs that had large somas, more than 4 primary dendrites and a “typical” type RI dendritic branching pattern (examples are shown in Fig. 4A). All RGCs from each treatment group were then plotted on separate scatterplots with the number of dendrites on the X axis and cell body size on the Y axis. RGCs that were identified as “RI-like cells” are shown as grey circles to identify them relative to the other cells (white diamonds; Fig. 4B). RI-like cells clustered together to the upper right of the scatterplot for all treatments, suggesting that they formed a coherent group. Most importantly, even though BDNF and CNTF RGCs had consistently larger cell bodies than the other treatment groups, the clustering was still apparent. To further confirm that our identification was robust, we performed a discriminant analysis on all RGCs, where cells were either classified as “RI” or “other”, and these cells were found to cluster into two distinct groups, regardless of treatment (Fig. 4C).

### Analysis of RI-like RGCs

**Figure 5:**
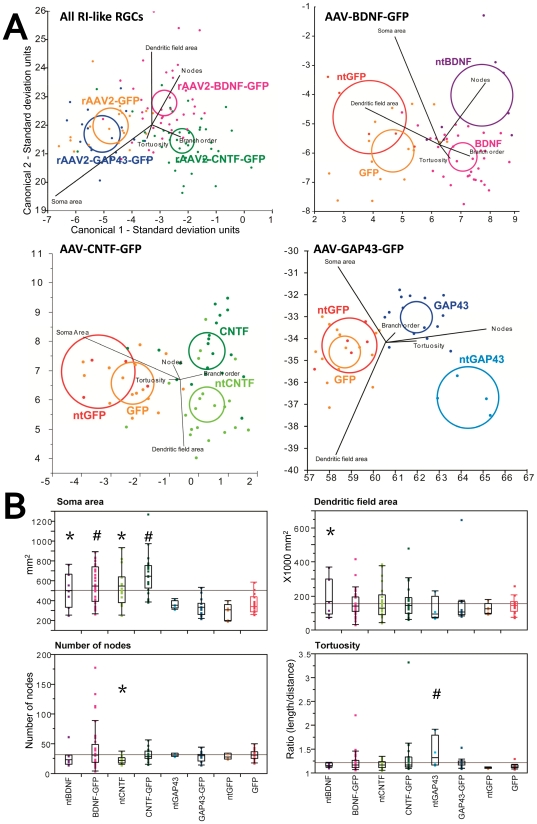
Evidence for morphological differences in type RI-like RGCs from retinae injected with rAAV2 encoding different transgenes. A: Plots showing canonical scores 1 (X axis) and 2 (Y axis) from multivariate discriminant analysis of dendritic morphology for RI-like retinal ganglion cells (RGCs). Plots show the first two canonical scores that together represent more than 80% of the variance. Axes represent units of standard deviation. Circles represent the 95% confidence region to contain the true mean of the group. Black lines show the coordinate direction (i.e. morphological parameters measured in Neurolucida) in canonical space. Note that the length of the lines is not representative of effect size due to the multidimensional nature of the analysis. B: Box plots showing median and quartiles for selected morphological parameters that were significantly different between treatment groups. Transduced and non-transduced (nt) FG^+^ RGCs in the 4 rAAV2 groups are labelled as GFP/ntGFP, CNTF/ntCNTF, BDNF/ntBDNF or GAP43/ntGAP43 respectively. Asterisk (*) indicates groups that are significantly different from ntGFP RGCs (p<0.05) and # indicates groups that are significantly different from GFP RGCs (p<0.05).

Plots showing canonical scores 1 (X axis) and 2 (Y axis) from multivariate discriminant analysis of dendritic morphology for transduced and non-transduced RI-like RGCs in each vector group are shown in Figure 5A; the data are summarized in Table 2. As seen in Figure 5B, BDNF and ntBDNF RI-like RGCs possessed soma areas that were significantly increased compared to GFP and ntGFP cells respectively (p = 0.04 for both). In ntBDNF RGCs, there was also a strong trend for a larger dendritic field area compared to ntGFP cells (p = 0.06; Fig. 5B). In addition, transduced and non-transduced RGCs in rAAV2-BDNF-GFP injected eyes were significantly different from each other, ntBDNF RGCs having a larger dendritic field area compared to BDNF RGCs.

In CNTF and ntCNTF RI-like RGCs, soma area was also significantly increased compared to GFP and ntGFP cells respectively (Fig. 5B). In addition, dendrites of ntCNTF RGCs were less complex than those of ntGFP cells, with fewer nodes and reduced branch order, as well as increased total and mean terminal/nodal distances, suggesting growth of terminal segments. As observed in rAAV2-BDNF-GFP injected eyes, transduced and non-transduced RGCs in rAAV2-CNTF-GFP injected eyes were significantly different; compared to CNTF RGCs, ntCNTF RGCs had significantly fewer nodes and lower order branching, confirming the loss of complexity in these non-transduced, or bystander, neurons.

Morphological differences in RI-like RGCs from rAAV2-GAP43-GFP injected eyes were attributed to an increase in tortuosity in GAP43 transduced cells compared with GFP transduced controls (Fig. 5B). Note here that Sholl analysis of RI-like RGCs failed to reveal any significant differences (data not shown).

### Stratification of dendritic tree

For the majority of FG^+^ RGCs, the dendritic tree extended within the inner half of the inner plexiform layer (on average within 30 µm of the cell body) suggesting that they were ON centre cells [40]. A small number of cells appeared to be either OFF or bistratified (ON/OFF) cells but these were not analyzed statistically due to low numbers (0–3 cells per treatment group).

**Figure 6:**
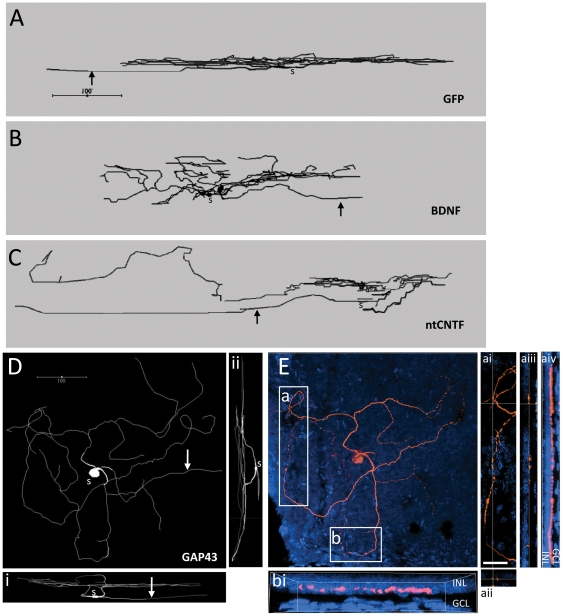
Abnormal stratification in RGCs from retinae injected with rAAV2 encoding different transgenes. A–C: Side views of Neurolucida traces showing examples of abnormal stratification in each rAAV2 treatment group. Arrows indicate axons and “s” is immediately below the soma. The scale bar in A applies to the three traces. D: Neurolucida trace of an rAAV2-GAP43-GFP transduced retinal ganglion cell (RGC). Ai and Aii are side views. E: confocal image of the same cell as in D; pink is Cy3 immunofluorescence for lucifer yellow, wholemount counterstained with Hoechst 33342 (blue). Areas a and b are shown in more detail. Area a is shown in panels ai-aiv. Panel ai shows a higher power magnification of a and panels aii and aiii show the XZ and YZ projections respectively at the crosshairs shown on panel ai. Panel aiv shows the full YZ projection of panel a and shows that dendritic processes descend through the inner plexiform layer and run along the border of the inner nuclear layer (INL), but they do not enter the INL. Area b is shown in panel bi as an XZ projection showing the relationship of this dendritic branch to the INL. GCL, ganglion cell layer. Scale bar in ai: 25 µm.

Within the population of presumed ON cells, sampled across a similar range of retinal eccentricities, the depth profile of dendritic trees was increased in all treatment groups, and this was mainly due to branches extending to an abnormal depth within the IPL (BDNF: p = 0.004; CNTF: p = 0.003; GAP43: p = 0.01; all compared to GFP controls Fig. 6A–C). BDNF affected only transduced RGCs (p = 0.049), CNTF affected both transduced and non-transduced neurons (p = 0.02 for both), and GAP43 affected only transduced RGCs (p = 0.002) compared to appropriate transduced or non-transduced GFP controls. In the population of RI-like cells, RGCs in rAAV2-BDNF-GFP injected eyes were not affected, whereas dendrites of rAAV2-CNTF-GFP transduced and non-transduced, and rAAV2-GAP43-GFP transduced RGCs ramified more deeply (p = 0.02; p = 0.03; p = 0.0009 compared to appropriate transduced or non-transduced GFP controls). In RGCs with grossly abnormal dendritic morphology, while dendritic branches were occasionally seen at the border of the inner plexiform and inner nuclear layers (INL), these processes were not seen to penetrate the INL itself (Fig. 6D, E).

## Discussion

The potential clinical benefits of using gene therapy to deliver growth factors to treat neurological or retinal dysfunction are currently under investigation [10], [13], [41]–[45]. Post-injury delivery of appropriate viral vectors may also be an effective treatment after neurotrauma [14]. Depending on circumstances, such therapies may need to be long term and it therefore seems prudent to examine whether there are any unintended consequences of sustained delivery of such factors on neuronal structure and function. We have shown previously that intraocular injection of rAAV-BDNF-GFP or rAAV-CNTF-GFP influences adult RGC survival and regeneration after injury [8], [9]. Here we show that, in FG^+^ RGCs that had regrown an axon into PN grafts and therefore had comparable access to factors expressed by the grafted PN tissue, intravitreal delivery of vectors encoding BDNF, CNTF or GAP43 nonetheless resulted in significant and complex changes in the dendritic morphology of the total population of regenerate neurons.

Each factor induced specific structural changes but overall, BDNF and CNTF increased RGC soma size, while BDNF increased dendritic field size and CNTF and GAP43 altered dendritic complexity. These changes did not obviously restore regenerating RGCs towards a more “normal” morphology, but rather added to the effects that were induced post-injury; thus BDNF further increased dendritic field size and CNTF reduced complexity in RGCs whose dendritic arbors had become larger [46] and less complex [37] following optic nerve lesion and PN transplantation. Furthermore, all three transgenes induced abnormal dendrite growth that was not restricted to normal sub-laminae within the IPL. A significant finding was that the morphologies of non-transduced (GFP^−^), FG^+^ RGCs in rAAV injected eyes were also differentially affected, thus the total impact of a given transgene is multifarious and extends to bystander neuronal populations [47].

As described in the Results, others have argued that it is possible to recognize and classify at least some rat adult RGC subtypes that are regenerating an axon into a PN graft [37], [38]. Regenerating type RI RGCs in particular appear to display the most characteristic and convincing similarities to this same class in normal retina. We identified all RGCs that had large somas, more than 4 primary dendrites and a “typical” type RI dendritic branching pattern and discriminant analysis showed that these cells were always clustered in a separate group, irrespective of vector treatment. The impact of rAAV-CNTF-GFP injections was most obvious in these RGCs, with a significant reduction in the complexity of the dendritic arbors. While additional phenotypic markers for RGC subtypes, other than morphology, would have been helpful in these experimental animals, few such markers are available for rat RGCs. Importantly, characterization of regenerating, type 1-like RGCs has been reported in other species [48]–[50] and in the cat a number of physiological studies on RGCs regenerating axons into PN grafts unequivocally confirms the phenotype of morphologically characterized alpha cells as being Y-type in character although with some altered receptive field properties [51], [52].

### Growth factors and dendritic morphology

#### BDNF

Studies of nervous system development and regeneration reveal pleiotropic effects of growth factors on dendritic morphology [17], [19], [39], [53], [54]. It is generally accepted that BDNF increases dendritic field size and complexity of branching [55], [56], although BDNF may also cause a loss of dendrite complexity [57], [58]. Consistent with these complex effects, we found that RGCs were differentially affected depending on whether RGCs were transduced or non-transduced. Dendritic field area was consistently increased in RGC within rAAV2-BDNF-GFP injected eyes, and was accompanied by increased dendritic length, specifically due to longer nodal segments, suggesting interstitial growth [46]. Perhaps surprisingly, the increased growth was not accompanied by changes in branch density, perhaps due to altered dynamics of loss and formation of dendritic branches; in *Xenopus* tadpoles, target-derived (tectal) BDNF increases, whereas local (retinal) BDNF decreases, RGC dendrite complexity [58]. The different effect of BDNF depending on whether it is local or target-derived is relevant because in the present study the adult RGCs regenerated into blind-ended PN grafts and thus were exposed to factors expressed in the grafts but they could not re-connect with central targets. The resulting loss of balance between retinal and target BDNF may have contributed to the non-characteristic growth patterns we observed.

A key finding was the effect of rAAV2-BDNF-GFP injections on both transduced and non-transduced, regenerating RI-like RGCs. Interestingly, the effects were most pronounced in the non-transduced, bystander population. BDNF secreted from single cells within brain slices of immature cortex has been shown to act as an “intercellular morphogen”, increasing dendritic growth in neighbouring neurons [16], [59]. There are many possible mechanisms whereby secreted, transgene-derived BDNF might exert its effects on RGCs: BDNF effects can be mediated by full-length (TrkB-FL) and truncated (TrkB-T1) receptors, and by the p75 receptor. TrkB-FL promotes dendritic growth and complexity via recruitment of PI3-kinase and perhaps MAP kinase signaling pathways [17], [56] although in our model TrkB-FL signalling may be less relevant because the receptor may have been down-regulated in response to sustained high levels of BDNF [60]. TrkB-T1 has slightly different effects than TrkB-Fl in that it increases dendrite growth in regions distal to the soma and inhibits proximal branching, at least in cortical pyramidal neurons [61]. In addition, BDNF signalling via p75 may also be involved because in hippocampal neurons, p75 overexpression reduces dendrite complexity [57] and NGF activation of p75 increases dendrite length [62].

#### CNTF

Sustained expression of CNTF in regenerating RGCs was associated with increased cell body size in all RGCs, but increased aberrant dendritic growth and a loss of dendritic complexity were detectable only in RI-like cells. Interestingly, changes in dendritic architecture were most pronounced in non-transduced RGC populations. Intravitreal delivery of rAAV2-CNTF-GFP results in extensive elongation of RGC axons [8], [9] but comparatively little is known about the impact of CNTF on dendritic architecture, although CNTF and leukemia inhibitory factor (LIF) have been reported to induce dendritic retraction in cultured sympathetic neurons [15]. The actions of CNTF, LIF and other cytokines are regulated by suppressor of cytokine signaling (SOCS) molecules and SOCS3 deletion enhances RGC axonal regeneration [63]. We previously reported that intravitreal CNTF injection results in a long lasting increase in SOCS3 expression in RGCs [64]. The more pronounced effects of rAAV2 mediated expression of the secretable form of CNTF on the dendritic morphology of non-transduced RGCs may reflect lower levels of SOCS expression in these bystander cells compared to transduced RGCs, the latter therefore having a reduced capacity to respond to the cytokine [24].

#### GAP43

AAV-GAP43-GFP expression primarily affected dendritic complexity and branching, and is consistent with the influence of GAP43 on cytoskeletal structure and neurite/axonal growth [65]–[67]. There is also direct evidence that motifs found in the GAP43 protein regulate dendritic growth and branching in cultured hippocampal cells [68]. We observed significant changes in all RGCs, characterized by the development of denser and more complex dendritic trees with more tortuous dendrites. It is unclear how vector induced GAP43 protein expression affected field density of non-transduced RGCs, given that the protein is not normally secreted. However, GAP43 may promote secretion of other factors that alter the growth of neighboring neurons. One candidate is the protease nexin 1 (PN-1), a serine-protease and thrombin inhibitor expressed in glia and neurons *in vivo* [69], [70]. Secreted PN-1 alters extracellular protease activity, influencing neuronal development [71] including neurite outgrowth [72]–[75]. GAP43 may influence PN1 secretion [76], potentially altering the retinal environment and contributing to the dendritic changes described here. Note that PN1 has also been implicated in pathological situations [70] and in Alzheimer's disease [77] where abnormal dendritic morphologies are common.

### Abnormal morphologies and stratification

Previous studies of RGC dendritic morphology following ON lesions with or without a PN graft have described a high proportion of “unclassifiable” cells [37]. Abnormal morphologies were most frequent in rAAV2-CNTF-GFP and rAAV2-BDNF-GFP injected retinae, suggesting that these two transgenes promote aberrant arborization and growth beyond what is normally observed following ON crush or PN transplantation [46], [51], [78].

Subsets of lamina-specified RGCs are tuned to distinct visual features [40], [79] and disruption to stratification leads to compromised visual function. As in the cat [52], [80], [81], the majority of regenerate RGCs possessed dendrites characteristic of ON-responsive cells, however we commonly observed inappropriate extension of dendritic branches into deeper regions of the IPL. Abnormal stratification was seen more frequently in rAAV2-CNTF-GFP and rAAV2-GAP43-GFP injected eyes and resembled the dendritic trees in retinae after exposure to increased glutamate levels [82], [83].

### Conclusions

While vector-mediated expression of secreted growth factors in neurons and other cells undoubtedly has beneficial effects on cell viability and regenerative growth, we now show that long-term overexpression of such transgenes alters the dendritic morphology of both transduced and non-transduced regenerating neurons, potentially altering the pattern and efficacy of the afferent synaptic input to these cells [84]. To determine whether similar transgene-induced changes are seen in normal RGCs we are currently completing a separate quantitative study examining changes in dendritic architecture of uninjured RGCs. Altered dendritic morphologies may affect the function of any conserved or reconstructed neural circuits, raising important questions for future study. In the visual system such changes may be a benefit or a hindrance to behavior; changes in dendritic field size and/or complexity in different RGC classes may increase detection capabilities in retinae with reduced RGC numbers but may also negatively impact upon visual acuity [52]. Altered dendritic architecture in other CNS regions would obviously be associated with different functional processing issues. While the time-course of the observed dendritic changes and how they relate to axonal regeneration remains to be determined, the present data suggest it is prudent to develop reliable systems that allow the effective regulation of transgenes, especially if gene therapy is to be used to provide neurotrophic support during the treatment and clinical management of neurodegeneration and neurotrauma.

## Materials and Methods

Data were obtained from female Wistar rats aged 8–10 weeks at the time of rAAV2 injection and PN-ON surgery. Rats were purchased from the Animal Resources Centre (WA) Experimental work was approved by The University of Western Australia Animal Ethics Committee and conformed to national NHMRC guidelines.

### AAV vectors

Each vector was commercially produced by GTC Virus Vector Core (NC, USA) and was generated from either the pTRUF12 plasmid (GFP, BDNF and GAP43) or the pTRUF12.1 plasmid (CNTF; gift of Prof. Joost Verhaagen). For bi-*cis*tronic rAAV2 vectors the relevant gene sequence also contained a post IRES site that also permitted subsequent GFP expression, hence resulting in the production of two individual proteins (the growth factor protein and the GFP protein as a marker for transduction). Expression was driven by the cytomegalovirus early enhancer chick-β-actin (CMV-CAG) promoter. The CNTF gene sequence included a nerve growth factor signal to allow secretion of the vector produced CNTF protein (gift of Prof. Sendtner). Due to the rAAV2 packaging size limitation this vector was based on the pTRUF 12.1 plasmid with a CMV-CAG promoter that lacked a promoter intron and thus provided sufficient space for the transgene [8]. Previous in vitro and in vivo studies using the CNTF, BDNF and GAP-43 rAAV-2 constructs have shown by Western blot, ELISA and immunohistochemistry that transduced cells express biologically active proteins and promote RGC survival and axonal regeneration [2], [8], [9], [85].

### Surgery

Rats were anaesthetized with an intraperitoneal (ip) injection of a 1∶1 mixture of ketamine (100 mg/ml) and xylazine (20 mg/ml; 1 ml/kg). For vitreal injections, 4 µl of either saline, rAAV2-GFP, rAAV2-BDNF-GFP, rAAV2-CNTF-GFP or rAAV2-GAP43-GFP (n = 5 per group) was injected into the temporal part of the left eye via a glass micropipette inserted just behind the ora serrata, the pipette tip angled in order to avoid damage to the lens. All vector concentrations were 1×10^12^ genome copies/ml. Seven to nine days later, rats were again anaesthetized (see above) and the ON was cut about 1.5 mm behind the eye and a segment of PN was grafted onto the stump to enhance regeneration [22], [23]. The graft consisted of a 1.5 cm segment of autologous tibial nerve sutured onto the proximal stump of the cut ON with 10/0 suture (Ethilon; Johnson & Johnson, Australia). The distal portion of the PN was positioned over the skull and the end sutured to connective tissue using 6/0 suture. Care was taken to avoid damaging orbital blood vessels and the ophthalmic artery lying beneath the ON; vascular integrity of the retina immediately after this procedure was confirmed in each rat. Animals received a subcutaneous injection of buprenorphine (0.02 mg/kg, Temgesic; Reckitt & Colman, UK) and an intramuscular injection of Benacillin (0.1 ml, Troy Laboratories Pty. Ltd. Australia).

### Retinal wholemount preparation

Grafted animals survived for 5–8 months before further analysis. The range of post-graft survival resulted from the fact that it was only possible to process a small number of animals at any given time. Intracellular RGC injections were done on 16 different days during that period. To control for any effect of post-operative survival time on RGC morphology, rats from different AAV groups were sampled across the 5–8 month range, 80% of animals sampled between 6 and 8 months after surgery. For example, PN grafted rats injected with rAAV2-CNTF-GFP (n = 5) were injected on weeks 26, 27, 30 (2 animals) or 33 after surgery while rAAV2-BDNF-GFP rats (n = 5) were injected on weeks 25, 26, 31, 32 or 33. Two to three days prior to sacrifice, rats were anaesthetized as above and RGCs were retrogradely labeled with 4% FG (0.5 µl) injected into the distal end of the PN graft, more than 1 cm beyond the suture point with the transected ON. Rats were sacrificed with pentobarbitone (150 mg/kg, ip) and the whole retina was rapidly removed from the eyecup in oxygenated AMES buffer and flat mounted RGC-side facing down onto a glass slide. A circle of black Millipore filter paper was lowered onto the retina. The tissue adhered to the filter paper and was turned over and placed RGC-side up in oxygenated AMES buffer.

### Single cell injections

The retina was placed in a slide chamber, immobilized with a small weight and superfused with oxygenated AMES for the duration of the experiment. Glass micropipettes (resistance: 50–300 MΩ) were filled by capillary action with LY (Molecular Probes/Invitrogen; 2% in 0.1 M Tris buffer). RGCs were injected with LY by inserting the micropipette into the cell soma under the control of a micromanipulator. Injections lasted for 2–5 min until dendrites appeared completely filled. The micropipette was slowly removed and the filled RGCs visualized and photographed under UV light and also at 488 nm to determine whether regenerate FG^+^ RGCs were transduced (GFP^+^) or non-transduced (GFP^−^) by the respective rAAV vectors (Fig. 1). We note that post-IRES GFP expression in AAV vectors can be lower compared to GFP driven directly by a promoter [86], thus it is possible that some apparently GFP^−^ RGCs were transduced but the GFP was not discernable. However, based on GFP expression we previously determined the transduction efficiency of our bi-*cis*tronic vectors in normal rat RGCs and found little difference between these vectors and AAV-GFP [8]. Furthermore, the present quantitative analysis consistently revealed significant differences between transduced and non-transduced RGC populations, thus we argue that if any RGCs were incorrectly characterized as non-transduced, their number was very small. Between 20–50 RGCs were intracellularly injected per retina (Fig. 1D). In all groups, RGCs were most easily visualized and injected at mid-retinal eccentricities (Fig. 1D), thus there was only minimal sampling from central or peripheral retina.

### Immunohistochemistry

At the end of each experiment, the retina was removed from the slide chamber, gently peeled off the filter paper and fixed in 4% paraformaldehyde for 2 hrs at room temperature in the dark. The fixed retinae were washed in PBS for 3×10 min and incubated overnight at 4°C with an anti-LY antibody (Santa Cruz Biotechnology), 1∶500 dilution in 1% triton-X-100, 1% BSA in PBS. Following a one hour wash in PBS at room temperature, retinae were incubated with a Cy3 goat-anti-rabbit antibody (Jackson Laboratories), 1∶300 in 1% triton, 1% BSA in PBS, for 4.5 hrs at room temperature in the dark. Retinas were then washed 3 times for 10 min in PBS and mounted in Citifluor, coverslipped and sealed with nail varnish. Slides were stored at 4°C until further analysis.

### Quantitative and statistical analysis of retinal ganglion cell morphology

RGCs were manually traced directly from immunolabelled retinal wholemounts using Neurolucida software and analyzed in Neurolucida explorer. The experimenter was blinded to treatment group. In all groups, RGCs were sampled from a similar range of eccentricities. Use of a digitized stage allowed 3-dimensional measurements to be taken directly from the tissue without confocal imaging. The resulting Neurolucida traces contained quantitative data in the Z-axis allowing an estimation of dendritic stratification depth. In selected RGCs with abnormal dendritic trees, stratification relative to the inner nuclear layer was visualized by counterstaining wholemounts with Hoechst 33342 (Sigma) dye. Dendrites were traced at 40× magnification under oil immersion (Uplan Apo 40×/1.00 oil Iris).

Due to various technical problems it was not possible to process retinae from 4 of the 25 PN-ON grafted eyes. In the remaining 21 retinae (4 saline, 4 rAAV2-GFP, 4 rAAV2-BDNF-GFP, 5 rAAV2-CNTF-GFP and 4 rAAV2-GAP43-GFP) a total of 500 cells in the ganglion cell layer were injected and their dendrites manually traced using Neurolucida. Of these, 125 (25%) were discarded, either because (i) after fixation and processing some were found not to be FG^+^ or did not have a clearly labeled axon projecting to the optic disk and therefore may not have been RGCs, (66 cells), or (ii) because the dendrites were extremely abnormal in appearance and it was not possible to be certain whether this was due to incomplete LY fills or genuine morphological changes (59 cells).

### Analysis of morphology

As previously described [37], we observed RGCs with morphologies comparable to those of normal intact cells [29], as well as RGCs with one or more abnormal structures including either very sparse dendrites or unusually tangled processes. To determine whether different transgenes were more likely to induce these abnormal morphologies, we compared the proportion of cells in each treatment group with abnormal dendrites using a Chi squared test.

To further characterize differences between RGCs we performed multivariate analysis (Discriminant analysis; JMP) of key morphological RGC parameters [87]. Parameters measured for each cell were: soma area, number of nodes, number of primary dendrites, total dendrite length, dendritic field area, average segment tortuosity, maximum order branching, fractal count, dendritic density (dendritic field area/total dendritic length), total terminal distance, average terminal distance, total nodal distance, average nodal distance, total terminal/nodal distance and average terminal/nodal distance.

A multivariate analysis was carried out to determine whether RGC morphology was significantly different between treatment groups. This analysis provided 4 canonical scores for each cell which were analyzed by ANOVA and confirmed to be significantly different between treatment groups (Tukey's post hoc test; p<0.0001 for all treatment groups for at least one of the canonical scores). Measurements of morphological parameters (eg. cell soma size, dendrite length etc) were then compared by ANOVA (Tukey's post hoc test; significant when p<0.05) to identify which parameters were responsible for the differences.

To determine whether transgenes had differential impact on transduced and non-transduced cells in each treatment group, transduced cells in experimental retinae were compared to transduced cells in rAAV2-GFP injected retinae and non-transduced cells in experimental retinae were compared to non-transduced cells in rAAV2-GFP injected retinae. Transduced and non-transduced cells were also compared within treatment groups. This analysis provided 3 canonical scores for each treatment, that were analyzed by ANOVA and post hoc tests (Tukey). If post hoc analysis of canonical scores revealed significant differences between groups, measurements of morphological parameters were then analyzed by ANOVA as described above.

Dendritic tree morphology was also assessed using Sholl analysis [27], which involves counting the number of dendrites intersecting concentric circles of increasing diameter (20 µm intervals) centered on the cell body. The radial distribution of dendrites was analyzed by ANOVA (repeated measures, Bonferroni post-hoc test). All 375 Neurolucida traces are available on the public data base NeuroMorpho.Org.
